# Children with Generalised Joint Hypermobility and Musculoskeletal Complaints: State of the Art on Diagnostics, Clinical Characteristics, and Treatment

**DOI:** 10.1155/2013/121054

**Published:** 2013-07-22

**Authors:** M. C. Scheper, R. H. H. Engelbert, E. A. A. Rameckers, J. Verbunt, L. Remvig, B. Juul-Kristensen

**Affiliations:** ^1^Education of Physiotherapy, Amsterdam University of Applied Sciences, Tafelbergweg 51, 1105 BD Amsterdam, The Netherlands; ^2^Department of Rehabilitation, Academic Medical Center, University of Amsterdam, Amsterdam, The Netherlands; ^3^Department of Rehabilitation Medicine, Maastricht University Medical Center, Maastricht, The Netherlands; ^4^Adelante School for Public Health and Primary Care, Maastricht University, The Netherlands; ^5^Education for Pediatric Physical Therapy, Avans University of Applied Sciences, Breda, The Netherlands; ^6^Department of Rheumatology, Rigshospitalet, Copenhagen University Hospital, Copenhagen, Denmark; ^7^Research Unit for Musculoskeletal Function and Physiotherapy, Institute of Sports Science and Clinical Biomechanics, University of Southern Denmark, Odense M, Denmark

## Abstract

*Introduction*. To provide a state of the art on diagnostics, clinical characteristics, and treatment of paediatric generalised joint hypermobility (GJH) and joint hypermobility syndrome (JHS). *Method*. A narrative review was performed regarding diagnostics and clinical characteristics. Effectiveness of treatment was evaluated by systematic review. Searches of Medline and Central were performed and included nonsymptomatic and symptomatic forms of GJH (JHS, collagen diseases). *Results*. In the last decade, scientific research has accumulated on all domains of the ICF. GJH/JHS can be considered as a clinical entity, which can have serious effects during all stages of life. However research regarding the pathological mechanism has resulted in new potential opportunities for treatment. When regarding the effectiveness of current treatments, the search identified 1318 studies, from which three were included (JHS: *n* = 2, Osteogenesis Imperfecta: *n* = 1). According to the best evidence synthesis, there was strong evidence that enhancing physical fitness is an effective treatment for children with JHS. However this was based on only two studies. *Conclusion*. Based on the sparsely available knowledge on intervention studies, future longitudinal studies should focus on the effect of physical activity, fitness, and joint stabilisation. In JHS and chronic pain, the effectiveness of a multidisciplinary approach should be investigated.

## 1. Introduction

In the last decade, increasing scientific research has focused on the diagnostics and consequences of generalized joint hypermobility (GJH) and joint hypermobility syndrome (JHS), as well as the consequences of hereditary diseases of connective tissue, including the most serious form of the Ehlers-Danlos syndrome, hypermobile type (EDS-HT) [1]. GJH is defined from a certain number of joint mobility tests [2] and is part of the diagnostic criteria for benign joint hypermobility syndrome (BJHS) [3], defined for adults, and for a number of serious hereditary connective tissue diseases, like Ehlers-Danlos Syndrome (EDS) and mild types of Osteogenesis Imperfecta. The criterion for GJH varies but has for adults been suggested to be at least 4 positive tests out of 9 using the Beighton's tests for joint hypermobility, equal to one of the major criteria in JHS. However, GJH can also be defined as at least 5 out of 9 using Beighton's test, as in EDS-hypermobile type (EDS-HT) [4]. For children GJH is mostly recommended to be at least 5 or 6 out of 9. The difference between GJH and JHS is, that JHS is a symptomatic GJH condition, while GJH is a nonsymptomatic condition [2]. In a recent review the prevalence for adults varies from 2 to 57% depending on age, gender, and ethnic origin [2]. For children the prevalence varies from 7 to 36%, primarily depending on the tests and criteria (especially the cut-off points) used for diagnosing GJH [5–8]. Children may experience a great variety of impairments as a result of increased laxity of the connective tissues. This not only affects physical fitness [9], motor development [10], and proprioception [11] but may also include problems with different organ systems [12] (e.g., skin, vessel, and internal organs) and psychological distress [13]. As a result, children may experience functional disability [14], which often presents difficulties in normal daily life. Increased pain intensity and decreased quality of life were reported in children with Joint Hypermobility Syndrome (JHS) [14]. All dimensions in the Pediatric Quality of Life Inventory module (QoL) were decreased compared to children without JHS, including physical and emotional functioning. This seemed to influence both social and school functioning, since these parameters in the QoL module also turned out to be poorer for the JHS versus the not JHS children. Other studies have reported proprioception and muscle torque deficits [11], which is also assumed to have a serious negative influence on activity as well as participation. GJH and JHS present clinicians and researchers with difficulties in providing and developing effective diagnostic procedures and multidisciplinary care. It requires a shared common language, as well as a shared framework for what constitutes a high quality intervention in terms of goals and implementation [15].

The International Classification for Child and Youth (ICF-CY) is a multidimensional model of functioning with participation as the key construct [16, 17]. This model provides a framework to describe limitations associated with a child's functioning and identifies influencing environmental factors. It has logical coherent content, aids in determining classification and effective decision-making, and is easily adopted in rehabilitation service [18]. Although the ICF-CY is frequently used in providing healthcare service and preventive care for children, it has not been described in children with GJH, JHS, and collagen diseases with GJH. In each chapter of the domains of the ICF-CY we have aimed at separating scientific knowledge on firstly children with GJH, and then JHS, in order to separate those with less from those with more severe conditions.

The aim of the present paper is to provide a state of the art of diagnostics and treatment of GJH and JHS in children and young adults, where the ICF-CY serves as a conceptual model. The rational for this is that diagnostics as well as treatment strategies in GJH and JHS as well as in collagen diseases with GJH are sparsely evidence based, which may either be due to misdiagnosing or delayed diagnosing [3]. This international group of authors is collaborating to lead research in the field of GJH and JHS. The paper was written using the current literature and up-coming future research as the foundation. To evaluate the outcomes of treatment strategies regarding randomized controlled trials in GJH, JHS, and collagen diseases with GJH, a systematic review was performed in Central and Medline, with critical appraisal and best evidence synthesis.

## 2. Generalised Joint Hypermobility and Benign Joint Hypermobility Syndrome 

Joint mobility is a continuous trait that varies with joint location and is strongly influenced by age, gender, and ethnic origin [2]. Regardless of the type of mobility (hypo-, normo-, or hypermobility) we presume that variation in mobility begins in utero as part of the individual's phenotype. Joint hypermobility has been known for centuries, but not until recently has it attracted a more profound and increasing scientific interest. The reason for this interest is probably an often observed concomitant presence of joint hypermobility and musculoskeletal pain, giving rise to the diagnosis benign joint hypermobility syndrome, defined for adults (BJHS) [19, 20], which may include more signs than just musculoskeletal complaints [3]. However, hypermobility and pain are also part of other syndrome criteria, such as the Villefranche criteria for the various types of Ehlers-Danlos syndrome (EDS) [4], conditions belonging to the group of hereditary diseases of connective tissues (HDCTs).

Although the authors are aware that the primary focus of paediatric physiotherapists regarding diagnostics and treatment strategies is the ICF-CY level of activity and participation, we present the current paper, including the literature associated with the classifications of function, activity, and participation. The problems at the level of function might explain the possible problems at the level of activity and participation and may guide treatment strategies. In physiotherapy, functional diagnostics and prognostics might be independent from clinical diagnosis. Therefore, GJH and JHS with unknown origin or due to collagen diseases might be treated in the same way they influence functional deficits.

## 3. Function (ICF-CY)

### 3.1. Generalized Joint Hypermobility

According to the American Academy of Orthopaedic Surgeons (AAOS), it is not possible to precisely determine mean joint mobility throughout the body [21]. Consequently, the AAOS developed consensus-based estimates in degrees derived from statistical means based on reports from four committees of experts. In general, joint mobility is regarded as a graded phenomenon [22], and a consensus has been developed that individual joint mobility follows a Gaussian distribution [23–25]. Abnormal joint mobility would reflect movements that deviate from the mean with ±2 standard deviations. However, for practical purposes, movement measurements (range of motion (ROM)) in degrees are not manageable when testing for GJH. Instead, the Beighton tests that apply a dichotomous principle are widely used [26]. The Beighton tests were described about 40 years ago [27], but only with photographs and short legends accompanying figures. A considerable variation exists in performance, at the cut-off level for a positive test and in the criteria definition of GJH [28].

The Beighton score, consisting of five clinical manoeuvres, is scored dichotomously (0/1) from which a total score, ranging from 0 to 9, is calculated. It is a widespread belief that GJH is present in adults with a Beighton score of ≥4, whereas other cut-off points for detecting the presence of GJH have been proposed (GJH ≥ 4, GJH ≥ 5, GJH ≥ 6, and GJH ≥ 7 [3–5, 29]). Although these testing procedures and diagnostic criteria have been in place for years and are considered the gold standard from infancy to old age [30], criticism has arisen from within the rheumatological and clinical genetic community about its diagnostic and clinical usefulness and predictive validity. Recently, standardized protocols have been described regarding the operationalization of the Beighton score [31]. Currently, the Beighton tests as well as the criterion for GJH have proved to have high inter-examiner reproducibility in children as well as in adults [5, 31, 32]. In adults and children the concurrent validity also seemed to be acceptable as the positive Beighton tests equal normal mean ROM + 3SD [13, 29, 31], and GJH also has a high correlation to a global joint index [29, 32]. Predictive validity of a different cut-off level for GJH has never been established.

### 3.2. Pain

GJH: generally, knee symptoms are described as the most frequently reported symptoms among subjects with GJH [11]. This is supported by a recent meta-analysis which reported that sports participants with GJH have an increased risk of knee joint injuries during contact sports but no altered risk of ankle joint injury [33]. However, an association between GJH and a history of glenohumeral joint instability has also recently been found [34].

Three population-based studies found that pain or dislocation/subluxation was not related to GJH5 or GJH6 in 8-year-old and 10-year-old children [7, 8], and in 7–15-year-old children with GJH [35].

Most of the studies describing associations between GJH and symptoms are cross-sectional [9, 11, 30, 36–39] and therefore not able to identify causal relationships.

JHS: it is not known why some children with GJH develop pain and other symptoms, while others do not. One study found that children 8-9 years old with JHS (for girls with a Beighton score ≥5, and for boys a Beighton score of ≥4) were more affected in other nonjoint related tissues (lower ultrasound values in bone, higher degradation products in urine, and higher skin extensibility) than a nonsymptomatic GJH group [12]. They also had higher values of total ROM. This could indicate a more systemic effect in children with symptomatic GJH. Compared with a healthy reference group, the nonsymptomatic GJH children still had larger total ROM and more profound skin extensibility [12]. Children at 9–12 years old with JHS and knee pain in the previous week further reported lower quality of life than healthy children at the same age [19]. One single 4-year follow-up study found GJH6 to be a significant predictor of recurrence of nonspecific pain in children at 14 years of age, especially in girls [6], and GJH6 was also a significant predictor of pain recurrence in the lower limb [40].

It has been suggested that GJH is associated with premature osteoarthritis [2, 41, 42], although no longitudinal study has shown such an association. Some of the mechanisms behind this association could be that an increased ROM may result in mechanical stress on parts of the cartilage ill-adapted to load, causing repetitive microtraumas, thereby promoting development of osteoarthritis [43–45]. Another explanation could be that GJH is an unknown connective tissue disorder which could be the main contributor to the development of osteoarthritis [43, 45]. A recent study found that adults with GJH walk with higher joint moments in the knees and hips, which may indicate an increased risk of developing osteoarthritis [46]. However, another study has suggested GJH to be a protective factor for osteoarthritis, since an inverse relationship was found between hypermobility and hand and knee osteoarthritis [47]. Furthermore, hypermobility was associated with lower serum cartilage oligomeric matrix protein levels, and genetic variations of this protein gene may account for some subgroups of JHS [47]. A recent study showed that children 8–16 years with JHS had significantly increased pain score, representing pain in the morning, daytime, evening, and at night, as assessed by a pain drawing [48].

### 3.3. Muscle Strength, Explosive Power

GJH: reduced total muscle strength was seen in nonsymptomatic 8- to 10-year-old children with GJH compared with a reference group [12]. Other case-control studies did not confirm reduced isometric or isokinetic knee extension and flexion in children with nonsymptomatic or symptomatic GJH [49, 50]. Neither was there reduced hamstrings-quadriceps (H/Q) ratio in children with GJH compared with a reference group; however, girls with nonsymptomatic GJH5 had reduced isokinetic normalised PT knee extension (eccentrically) [50]. 

JHS: Children with JHS between 9 and 13 years of age had reduced isometric knee extension and flexion peak torque (PT) compared with a healthy control group [11].

### 3.4. Proprioception

GJH: in young adults with GJH no differences were found regarding joint position sense of the shoulder joint, nor differences in reflex latency of upper and lower trapezius compared with a healthy control group [51, 52].

JHS: in children 9–13 years of age with JHS (GJH 6, multiple joint pain, actual/historical), a decreased proprioception was found during knee extension, measured as passive joint position sense and threshold for detection of a passive movement [11]. This was partly confirmed in another study of adolescents and adults with JHS where the reflex in the knee extensors was absent in 47% of 15 patients, compared with a healthy control group in which this reflex was present in all subjects [52].

### 3.5. Balance

GJH: in a population-based study, children with GJH5 at 8 years of age had significantly better static balance in “stork stand.” Furthermore, they had the same dynamic balance, measured as agility, as the children without GJH [7]. Similarly, 10-year-old children with nonsymptomatic GJH performed just as well as a matched healthy control group in dynamic one-board balance [8].

In a case-control study, 8-year-old children with GJH5 did not have increased muscle steadiness (poorer precision) during submaximal knee flexion and extension (25% MVC for 15 seconds) compared with children without GJH. However, even though there was no significant correlation between steadiness during knee flexion and extension and Beighton score, steadiness was significantly worse in the children's parents with GJH4 [49]. These nonsymptomatic children with GJH5 further presented with decreased muscle activity in their knee flexor muscles (m. biceps femoris, m. semitendinous) during submaximal knee flexion (25% MVC), and an increased cocontraction during knee flexion [48]. This was not seen in the knee extensor muscles (m. vastus lateralis, m. vastus medialis) during knee extension. This illustrates a possible changed muscle activation pattern in knee flexion in children with GJH5 [49]. A recent study of dynamic balance showed that during gait, 10-year-old children with GJH5 walked with a less flexible movement pattern, that is, reduced lateral head stability and increased lateral head ROM, in addition to less stable trunk segments during enhanced balance challenges. The long-term consequences of this abnormal movement pattern are, however, unknown [53].

JHS: a recent study showed that children 8–16 years with JHS had significantly decreased balance, as assessed by the Bruininks-Oseretsky test of motor proficiency [48].

### 3.6. Extra-Articular Features

Numerous extra-articular features have been associated with GJH, such as chronic constipation and encopresis, enuresis and urinary tract infections, chronic fatigue syndrome, temporomandibular joint disease and fibromyalgia, although there is no universal agreement on the causal relationship [54].

## 4. Activities (ICF-CY)

### 4.1. Motor Development

GJH: several studies indicated that children with GJH have delayed and/or insufficient motor competence, described as clumsiness in early childhood and poor coordination [9, 55, 56]. One study reported that in children without GJH any delayed motor development disappeared before the second year of age, whereas in children with GJH, it has been reported to continue [57, 58]. Newer population-based studies did not confirm this association between motor deficits and GJH (with and without symptoms) [5, 7, 41], and in children between 7 and 15 years of age, Beighton score correlated negatively with disability, meaning that children with GJH did not experience functional limitations in daily activities [35]. In fact, nonsymptomatic children with GJH5 and GJH6 performed better in a finger speed and reaction test [7] and so did the boys with GJH6 in a finger coordination test [8].

JHS: in a population of children who were referred to a university hospital, severe delays in motor development were observed in approximately one third of the children with JHS, whereas there was no association between the level of GJH and delay in motor development [10].

Few objective physical performance and capacity measures have been used to assess physical function in children with GJH, not even as outcomes in the few treatment studies of children with GJH. This is somewhat surprising, since the interventions all included elements of physical exercise and performance [59, 60]. Instead primary outcomes in these studies are mostly self-reported disability and pain, in addition to injury prevalence, while physical performance outcomes such as muscle strength, endurance, balance, proprioception, motor control, posture awareness, or stability are not measured [59–61], or even recommended to be measured [62].

### 4.2. Gait Pattern

GJH: in a case-control study, children with GJH5 walked with significantly decreased peak moments in their knee (flexion moment at heel strike and in mid-stance, and knee abduction moment in early push-off phase), and in their hip (extension and abduction moment) but an increased dorsiflexion moment of the ankle. The ankle kinematics differed significantly between groups, while there was no difference in the knee and hip kinematics [63]. However, the clinical relevance needs to be determined through longitudinal investigations.

JHS: as far as the gait pattern of children with JHS is concerned, it has been written that “a combination of hypermobile joints, reduced proprioception, weak muscles, and reduced stamina (endurance) can profoundly affect the gait of a child with JHS”. To correct this, the causes of the abnormalities need to be identified and worked on separately, before the gait will improve [38]. However, few studies have actually shown this abnormal gait pattern in gait analyses. In one study, children with JHS (GJH ≥ 6), and multiple joint pain (currently or historically in more than one joint) had a significantly lower peak knee flexion angle both during the loading response and the swing phase, as well as an increased knee extension in mid-stance during walking compared with a matched group of healthy children, while there was no difference in gait speed between the groups [63].

### 4.3. Vertical Jump

GJH: children (10 years old) with GJH5 had significantly higher peak vertical jump height, without a significant correlation between Beighton score and jump height, while there was no higher rate of force development in GJH5 [50]. A similar result was found in girls with GJH6 who had an insignificantly higher vertical jump height compared with girls without GJH, and significantly positive correlations between Beighton score and vertical jump height [8]. Further studies on this aspect are necessary to confirm these findings, that nonsymptomatic GJH may produce more explosive power than children without GJH, and to judge the practical implications of this, for example, as an increased risk for future injuries.

### 4.4. Physical Fitness

GJH: children with GJH did not spend fewer weekly hours of physical activity than healthy reference groups [7, 8, 12, 64]. This fits well with the hypothesis that having nonsymptomatic GJH may even be an advantage for selection into certain elite sports, such as ballet, dance, and gymnastics, due to the capacity for increased range of movement [5, 64, 65]. Consequently, general physical fitness must be adequate in children with nonsymptomatic GJH, in order to be selected into elite sports. A recent population-based study of 6022 children at 14 years of age found a positive association between girls with GJH and physical activity, measured with accelerometry [66]. A similar trend was seen in a school population of 7–15-year-old children, where hypermobile children were slightly more active than nonhypermobile children, based on self-reports [35]. 

JHS: however, many hours of physical activity need not be closely related to high endurance and/or aerobic fitness. This poor relationship was seen in children (6–20 years) with JHS and exercise-induced pain and intolerance who actually had reduced aerobic fitness compared with a healthy reference group, measured as absolute and relative (related to body mass) peak VO2 [64]. The reason for this poor aerobic fitness was assumed to be due to musculoskeletal pain, resulting in inactivity and deconditioning, which could then result in exercise-induced pain and intolerance [64].

### 4.5. Participation (ICF-CY)

JHS: children with JHS are less active in sports and miss education more often in comparison with their healthy peers with normal joint mobility [56]. A recent study showed that children 8–16 years with JHS had significantly decreased participation in housework, riding a bicycle, taking part in sport or outdoor games, as assessed by the Frequency of Participation Questionnaire [48]. Also a higher frequency for participating in nonsporting games besides a higher need to rest was reported in children with JHS [48].

Personal communication and case reports mention the influence of GJH and JHS on participation, whereas the environmental and personal factors of children with GJH and JHS have been anecdotally reported, for example, the role of family dynamics and coping with JHS.

## 5. Treatment Strategies

In general, knowledge of GJH and JHS is clinically important and tailored care should be based on the individual's complaints and needs. Tailored care in terms of evidence-based diagnostic procedures and clinical expertise is essential in order to construct classification models as well as optimized treatment strategies. So diagnostics and subsequent treatment should be determined from evidence-based practice, clinimetrics, and clinical reasoning, eventually leading to classification and treatment strategies.

Reassurance, education, and joint care are cornerstones of treatment strategies [54]. In children and adults, uncontrolled studies and therapeutic strategies have been described [62, 67, 68]. In a recent review Keer and Simmonds described that joint protection and injury prevention form a major component of a successful rehabilitation programme. The aims are achieved through improving posture, joint stability, and specific motor skills, which include pain-free, cognitive exercise to enhance proprioception and muscle strength. Renewed confidence in one's own joints will lead to a resumption of a more normal level of physical activity with the benefits of improved physical fitness and wellbeing. The optimal form of rehabilitation to maintain joint health in JHS is, however, questioned [68]. Since observations are primarily based on noncontrolled trials, we have to be cautious with the interpretations of such literature. 

A systematic review was conducted to find randomized controlled trials focusing on interventions for GJH, JHS, and GJH in collagen diseases like EDS, Osteogenesis Imperfecta, and Marfan. Publications were retrieved from the bibliographic databases CENTRAL (searched in the Cochrane library) and MEDLINE (searched in PubMed). In PubMed, only MeSH terminology was applied combined with the sensitive Cochrane filter for reviews on interventions and limited to humans. In CENTRAL, only free-text terms were applied. The following keywords were combined and used: GJH, JHS, Ehlers Danlos, Osteogenesis Imperfecta (OI), Marfan Syndrome, child, treatment, and rehabilitation. From these keywords MeSH headings were derived and inserted into the electronic search. The references of retrieved trials and other relevant publications including reviews and meta-analyses were examined (cross-referencing). The following criteria were used for including studies: (1) only patients with musculoskeletal complaints, in terms of pain and fatigue, diagnosed with GJH, JHS, Ehlers-Danlos Syndrome (all types), Osteogenesis Imperfecta (all types), or Marfan Syndrome were included in the study, (2) all studies should only focus on the effect of treatment in children, between the ages of 0 and 18 years of age, (3) only physical and cognitive oriented treatment modalities should be evaluated: medicinal, surgical, or treatment by assistive devices were excluded, (4) only English publications were considered for inclusion: letters, dissertations, abstracts, and case studies were excluded.

## 6. Critical Appraisal of Included Studies

Two assessors (MCS and RHHE) performed retrieval of studies for the present review. The two assessors independently evaluated the identified publications, classified the identified studies according to predetermined criteria, and reviewed the methodological quality of each study, using the Physiotherapy Evidence Database (PEDro) methodological scale [69]. The PEDro scale was developed for rating quality of RCTs and contains 11 items. The first item represents external validity of the trial. This item is not included in the total PEDro score (maximum 10); therefore, our score is based on items 2 to 11. These items represent 2 aspects of trial quality, the internal validity of the trial and whether the trial contains sufficient statistical information. These items are scored either yes (1 point), no, or not applicable (0 points). The individual item scores and the total PEDro scores have been shown to be reliable [70]. Studies with PEDro scores of 4 points were classified as “high quality,” whereas scores of 3 points and below were classified as “low quality” [71]. During a consensus meeting, scoring disagreements were resolved. In the event that agreement could not be reached, a third reviewer (BJK) decided on the final score. Reviewers were blinded to author(s), institution(s), or journals.

## 7. Best Evidence Synthesis

**Figure 1:**
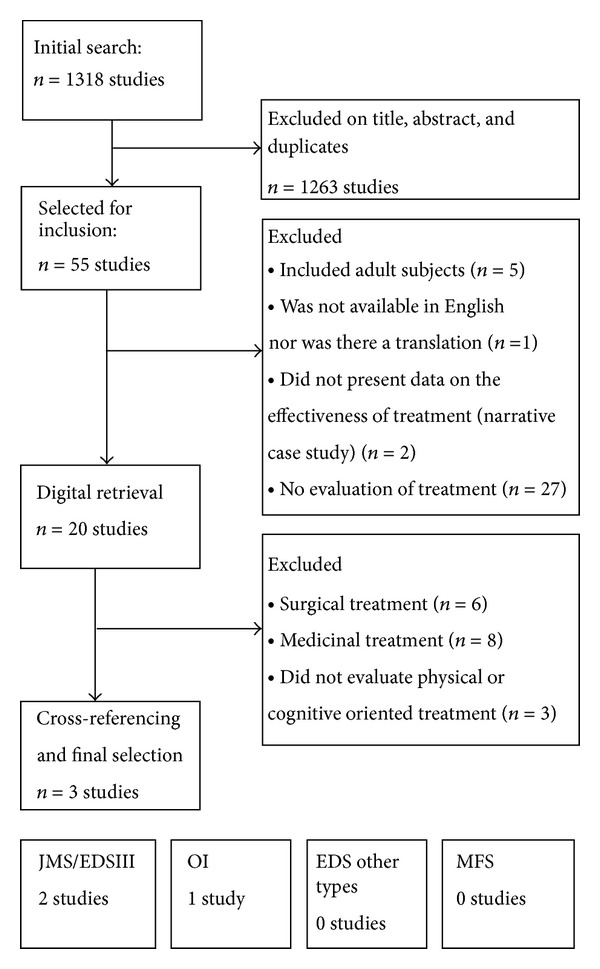
Flow diagram of the selection process of included studies.

Statistical pooling of the included studies was not feasible due to methodological heterogeneity in interventions, patient characteristics, and outcomes. Therefore a best evidence synthesis (BES) was applied based on the criteria of van Tulder et al. (Table 1: best evidence synthesis) [72]. These criteria are based on the PEDro scale. Selected studies were categorized into 5 levels of evidence: (1) strong evidence; (2) moderate evidence; (3) limited evidence; (4) indicative findings; and (5) no or insufficient evidence (Table 1) [72]. The initial search identified 1318 studies (Figure 1). After selection on title and abstract 55 studies were selected for further scrutinizing. After the first selection round, 35 studies were rejected for the following reasons: 27 studies did not evaluate treatment effects, 2 studies did not present data on the effectiveness of treatment (narrative case study), 5 studies evaluated treatment in adult subjects, and 1 study was not available in English or could be translated. In total, 20 studies were digitally retrieved and were cross-referenced for additional potential studies. Cross-referencing identified 1 additional study. In the final selection round 17 studies were further rejected on the bases of the following: 6 studies evaluated treatment effects of surgical interventions or the use of assistive devices, 8 studies focused on medicinal treatment, and 3 studies did not evaluate treatment modalities including a physical or cognitive approach. That left the present review with 3 studies (JHS/EDS hypermobile type: *n* = 2, OI: *n* = 1) available for critical appraisal [59, 61, 73].

## 8. Critical Appraisal

Initially, disagreement was present in 5 out of 60 scored items (8,3%), but after discussion total agreement was established. The total PEDro score of the included studies ranged from 3 to 8 and included a total population of 95 children (Table 2). All included studies were of RCT design in which independent allocation was secured. However, in the study of Mintz-Itkin et al. [61], no randomization method was specified and thus was found to be questionable. Only in the study of Mintz-Itkin et al. baseline comparability was established, while in the study by Kemp et al. no statistical data was presented in which baseline comparability was ascertained [59]. In all included studies no double-blinding was applied; however, in the study by Kemp et al. therapists were blinded for subject characteristics and disease characteristics. Only in the study of van Brussel et al. blinding of outcome assessors was applied, while it was unclear in the remaining studies. Loss to follow-up was reported in the study of van Brussel et al. [73] and in the study of Kemp et al. [59]. However the study by Kemp et al. failed to report of >85% of the initially included population but did show the absence of selective loss to follow-up. The study by Mintz-Itkin et al. did not provide complete descriptions of loss to follow-up [61], and no intention-to-treat analysis was performed. However, in van Brussel et al. [73] and Kemp et al. [59], all analyses were performed by intention-to-treat. 

## 9. Best Evidence Synthesis

Two studies were included evaluating the effectiveness of enhancing physical fitness in children with OI and JHS (Table 3) [59, 73]. Both studies had PEDro scores of ≥6 (range: 6–8) and were classified as high-quality RCT studies, including 91 children between the ages of 7 and 18. Both studies showed significant benefits of enhancing physical fitness in terms of relieving musculoskeletal complaints (pain and fatigue) and reducing disability. Based on the best evidence strategies there is strong evidence that enhancing physical fitness in children with connective tissue diseases is effective. Two studies evaluated treatments focusing on enhancing motor control in children with GJH and JHS/EDS-hypermobility type (Table 3). Due to the large difference in age (infants versus children and adolescents), no BES was classified for the evidence of enhancing motor control.

## 10. Discussion

Observational studies have been performed to describe the clinical characteristics in GJH and JHS. Whether GJH and JHS are separate or related entities is not clear, since prospective follow-up studies in GJH children leading to JHS are scarce. Although many case reports have been written regarding interventions for GJH and JHS, few randomized controlled trials have been performed. Future randomized controlled trials are indicated to study the effects of stand-alone (an)aerobic, strength and stability, as well as coordination training in different combinations. Until these findings have been published it will not be possible to develop evidence-based treatment protocols and guidelines for training JHS. These interventions may be based on the training principles for healthy children and children with chronic diseases. Over the last decade, children's training has been discussed from the perspective of physical fitness in physical therapy. The focus on physical fitness has increased, due to the decreased physical fitness of normal developing children [74], along with the effects of training on children with different diagnoses [75]. Both cardiovascular and strength training are important aspects of physical fitness. Guidelines for cardiovascular and strength training in children are described in Strength & Conditioning Professional Standards and Guidelines and the Position Statements published by the National Strength and Conditioning Association (NSCA) [76, 77]. In the guidelines for strength training (NCSA), the training principles are described: the frequency of training 1–3 times a week, for at least 8 weeks, but preferably 12 weeks, the advised load (repetition maximum (RM)) 8–12 RM, and the preferable method Progressive Resistance Training (PRE) [78]. Strength training should be performed only after the age of 7 years and ideally under supervision. 

In a recent systematic review, the effectiveness of proprioceptive and coordination training in preventing sports injuries was described [79]. Results of seven methodologically well-conducted studies were presented. Multi-intervention training was effective in reducing the risk of lower limb injuries, acute knee injuries, and ankle sprain injuries. Balance training alone resulted in a significant risk reduction of ankle sprain injuries [79]. However, the effect of this training on children as well as adolescents with JHS is not known. It is concluded in the NCSA position statements that school-aged youth should participate daily in 60 minutes or more of moderate to vigorous physical activity that is enjoyable, appropriate to their stage of development, and involve a variety of activities. Not only is regular physical activity essential for normal growth and development, but also a physically active lifestyle during the pediatric years may help to reduce the risk of developing some chronic diseases later in life. Resistance training can offer unique benefits for children and adolescents when appropriately prescribed and supervised. Combined comprehensive school-based programmes are specifically designed to enhance health-related components of physical fitness including muscular strength [76, 77]. 

JHS: based on the literature, it would be ideal if a randomized controlled trial was performed in JHS children, assessed with instruments with high psychometric qualities, and also with objective measurements, with an intervention period of at least 12 weeks and a progressive training intervention of a minimum of 3 times a week, in which the dose of training intervention is effective, based on the above-mentioned training principles. 

GJH: little is known about the children with GJH who develop eventually JHS. Although pain is more common in children and adolescents with GJH as compared with their nonhypermobile peers, only a minority of children with pain will eventually develop a chronic pain syndrome. However, for these children, pain can have a huge impact on their life and development, interfering with school and leisure time activities. Chronic pain syndromes, like complex regional pain syndrome [80] or chronic widespread pain syndrome [6, 81], have increasingly been associated with underlying ligamentous laxity in both adult and paediatric populations. 

A traditional biomedical approach with a single focus on physical impairment is in most cases insufficient to explain the total impact of chronic pain and its associated disability. Studies among children with chronic pain [82] suggest that behavioural and psychosocial factors contribute to the development and maintenance of chronic pain. Recently, increasing evidence came available confirming a central role to the concept of pain-related fear in children/adolescents with chronic pain [10, 83]. In the fear avoidance model, one of the most prominent explanatory models for disability in pain research, it is stated that a subgroup of persons will, after an acute pain problem, interpret their pain as threatening [83]. For these individuals, expectations of adverse consequences of physical activity, such as a further increase in pain or (re)injury, will be a reason for avoiding physical activity [84]. Over the long term, avoidance behaviour will thus result in a combination of negative health consequences: disability, depression, and disuse, the last of which may be defined as a decreased level of physical activity in daily living [85]. In adults, numerous studies have confirmed the role of fear of pain/reinjury in explaining pain related disability [86]. In children and adolescents, recently more evidence came available confirming the applicability of the fear avoidance model in children/adolescents [83, 87]. Parent perceptions of, and responses to, pain have been identified as important additional factors contributing to pain-related disability among children and adolescents with chronic pain [88]. 

Whether fear of movement/(re)injury accounts for an additional disabling influence in children and adolescents with disabling GJH is currently still unknown. However, the high incidence of GJH in paediatric populations and the need for multidisciplinary care for their pain problem [38] seem to indicate the multidimensionality of their pain problem. It can be hypothesized that, in children with JHS, the role of fear of movement is even more pronounced than in children with pain without hypermobility, since recent studies have shown that JHS appears to be associated with a higher risk of developing anxiety disorders [86]. And since previous research has shown that anxiety sensitivity is strongly associated with fearful appraisals of pain, the occurrence of pain related fear in JHS can be expected to be higher as compared to that in children/adolescents with pain without hypermobility [89].

As a consequence, an acute pain problem, such as a musculoskeletal complaint or (sub)luxation in a child with GJH may therefore have a disabling impact on its own, but, in those with a high level of pain-related fear, it may also lead to a downward spiral by avoiding further activities. Since the JHS has been indicated as one of the most frequent causes of musculoskeletal symptoms in children and adolescents aged between 13 and 19 years of age [90], this population seems more prone to developing chronic pain syndrome as compared with those children without JHS.

A recent systematic review identified a positive effect of behavioral treatment in children with chronic pain as a single psychological treatment [91]. For those with disabling chronic pain, currently no studies in a randomized design are available to confirm the effect of multidisciplinary treatment, although studies in a prepost design do support its value [92]. In addition, whether the most effective treatment for children and adolescents with GJH and JHS would be graded exposure treatment, which is a multidisciplinary treatment specifically targeting pain and disability-related problems, is currently still unclear.

## 11. Conclusions

Generalised joint hypermobility (GJH) with and without musculoskeletal complaints is frequently observed in children and young adults. Based on a narrative and a systematic review, knowledge on function and activity in GJH and JHS is available, and knowledge on participation, personal and environmental factors recently showed a significantly decreased participation in housework, taking part in sport or outdoor games, as well as a higher frequency for nonsporting games.

If and why children with GJH eventually develop JHS is not known, due to lack of prospective, longitudinal studies.

Based on the sparsely available knowledge of intervention studies, future longitudinal studies should focus on the effect of physical activity and fitness, as well as muscle strength and stabilisation in general, and in the hypermobile joints in particular. In JHS and chronic pain, the effectiveness of a multidisciplinary approach should be investigated. 

## Figures and Tables

**Table 1:** Best evidence synthesis.

Level of evidence	
Strong evidence:	Provided by statistically significant findings in outcome measures in at least 2 high-quality RCTs with PEDro scores of at least 4 points

Moderate evidence:	Provided by statistically significant findings in outcome measures in at least one high-quality RCT and at least one low-quality RCT (3 points on PEDro or one high-quality CCT)

Limited evidence:	Provided by statistically significant findings in outcome measures in at least one high-quality RCT or at least 2 high-quality CCTs (in the absence of high-quality RCTs)

Indicative evidence:	Findings provided by statistically significant findings in outcome measures in one high-quality CCT or low-quality RCTs (in the absence of high-quality RCTs), or 2 nonexperimental studies of sufficient quality (in the absence of RCTs and CCTs)

No or insufficient evidence:	In the event that results of eligible studies do not meet the criteria of one of the previously stated levels of evidence, or in case of conflicting (statistically significant positive and statistically significant negative) results among RCTs and CCTs, or when no eligible studies are available, CCTs indicates controlled clinical trial.

**Table 2:** Study characteristics and study results.

Author (year)	Diagnosis (age, range in years)	Sample size	Design/time-intervals (weeks after *T*_0_)	Experimental treatment (*w*/*f*/*i*)	Control treatment (*w*/*f*/*i*)	Outcome domains (ICF-CY)	Results	Authors conclusions
van Brussel et al., (2008) [73]	OI (8–18)	*N* = 34 (E: 16/C: 17)	RCT *T*_1_: 12 *T*_2_: 24 *T*_3_: 36	Physical training (12/3/45)	Usual care (?/?/?)	(i) Physical fitness (ii) Fatigue (iii) Perceived competence (iv) HRqOL	Improvements were found on all outcomes in favor of E at *T*_1_. At *T*_2_ and *T*_3_ scores deteriorated	Supervised program improves physical fitness and reduces fatigue safely and effectively

Mintz-Itkin et al., (2009) [61]	GJH (0-1)	*N* = 29 (E: 15/C: 14)	RCT *T*_1_: 36 *T*_2_: 48 *T*_3_: 60 *T*_4_: 72	Monthly, Bobath treatment (?/?/?)	Weekly, Bobath treatment (?/?/?)	(i) Gross motor development (ii) Achievement motor mile-stones	Motor catch-up was achieved in both groups, (no significant between-group difference)	Monthly physical therapy combined with home exercises is sufficient to achieve motor catch-up

Kemp et al., (2009) [59]	JHS/EDSIII (7–16)	*N* = 57 (E: 30/C: 27)	RCT *T*_1_: 6 *T*_2_: 12	Enhancing joint control of symptomatic joints (6/1/30)	Physical training (6/1/30)	(i) Physical fitness (ii) Pain scores (iii) Disability	Both groups improved in perceived pain and functional ability, (no between-group difference)	Both interventions demonstrated significant pain reduction, (no between-groups difference)

OI: Osteogenesis Imperfecta, GJH: generalized joint hypermobility, JHS: joint hypermobility syndrome, EDSIII: Ehlers-Danlos hypermobile type, E: experimental group, C: control group, RCT: randomized clinical trial, *w*: weeks of treatment, *f*: frequency per week, *i*: intensity in minutes per session, ICF-CY: International Classification of Functioning for Child and Youth.

**Table 3:** Critical appraisal of included study designs, risk of bias.

		Items											Remarks
		Treatment allocation			Blinding			Loss to follow-up				Total score	
Study	Eligibility criteria	Randomized	Concealed allocation	Baseline comparability	Subjects	Therapists	Assessors	Measures obtained in 85% of all initially included subjects	Intention to treat	Group comparison reported	Point measures and measures of variability reported		
van Brussel et al., (2008) [73]	Yes	Yes	Yes	Yes^1^	No	No	Yes	Yes	Yes	Yes	Yes	8/10	^ 1^Corrections were applied

Mintz-Itkin et al., (2009) [61]	Yes	No^2^	No^2^	Yes	No	No	No	No^3^	No	Yes	Yes	3/10	^ 2^No randomization method was specified ^3^No data on loss to follow-up was presented

Kemp et al., (2009) [59]	Yes	Yes	Yes	Yes^4^	No	No^3^	No	No	Yes	Yes	Yes	6/10	^ 3^Therapists were blinded for disease characteristics ^4^No formal statistical data was presented in which baseline comparability was ascertained

## Abbreviations

JHS: Joint hypermobility syndrome
EDSIII: Ehlers-Danlos hypermobile type
OI: Osteogenesis Imperfecta
EDS: Ehlers Danlos
MFS: Marfan syndrome.
